# R3Net: Recursive Residual Refinement Network Architecture for Decoder‐Free Medical Image Segmentation

**DOI:** 10.1002/pro6.70082

**Published:** 2026-07-31

**Authors:** Jing Huang, Yongkang Zhao, Yuhan Li, Zhitao Dai, Cheng Chen, Qiying Lai

**Affiliations:** ^1^ School of Computer Science and Artificial Intelligence, Wuhan University of Technology Wuhan China; ^2^ National Cancer Center/National Clinical Research Center for Cancer/Cancer Hospital & Shenzhen Hospital, Chinese Academy of Medical Sciences and Peking Union Medical College Shenzhen China; ^3^ Department of Radiation and Medical Oncology Zhongnan Hospital of Wuhan University Wuhan China

**Keywords:** Decoder‐free Architecture, Medical Image Segmentation, Multiscale Features, Recursive Residual Refinement

## Abstract

**Background:**

Medical image segmentation methods based on encoder–decoder architectures often achieve high accuracy but typically require substantial computational resources and contain redundant parameters.

**Purpose:**

This study aims to develop an efficient decoder‐free segmentation framework that maintains competitive performance by strengthening the encoding process.

**Methods:**

We propose R3Net, an encoder‐only segmentation architecture based on a recursive residual refinement (R3) mechanism. By recursively reusing encoder stages and progressively fusing multiscale features through residual pathways, R3Net reconstructs high‐resolution features without requiring a dedicated decoder.

**Results:**

Experiments on three medical imaging modalities—cardiac MRI (Automated Cardiac Diagnosis Challenge), abdominal CT (Synapse), and thyroid ultrasound (Thyroid Nodule Multimodal Learning)—demonstrate that R3Net achieves segmentation performance comparable to representative encoder–decoder models while reducing the number of model parameters and computational complexity.

**Conclusion:**

R3Net provides an effective decoder‐free alternative for medical image segmentation, suggesting that competitive dense prediction can be achieved through recursive refinement within the encoder.

## Graphical abstract

We propose R3Net, a decoder‐free medical image segmentation framework that recursively refines multiscale representations within the encoder using residual pathways. R3Net achieves competitive accuracy with reduced model complexity and improved computational efficiency across multiple medical imaging modalities.

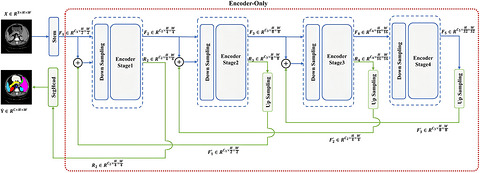

**Abbreviations**
ANA: antinuclear antibodies
APC: antigen‐presenting cells
IRF: interferon regulatory factor.

## 1 INTRODUCTION

The encoder–decoder architecture with skip connections is widely adopted in many computer vision tasks. In particular, it has become a dominant paradigm in modern medical image segmentation. Beginning with the classic UNet,^1^ numerous segmentation models have subsequently been proposed following the UNet–like architecture, such as nnUNet,^2^ TransUNet,^3^ Swin‐UNet,^4^ and MSVM‐UNet.^5^ This architecture typically employs a pyramid‐structured encoder to capture hierarchical, multiscale feature representations. During encoding, the channel dimensionality of feature representations gradually increases with encoder depth, whereas their spatial resolution decreases stepwise. Meanwhile, the decoder progressively upsamples the features extracted by the encoder and refines them to produce high‐resolution dense predictions with the aid of skip connections. As shortcut connections bridging the corresponding encoder and decoder layers, skip connections help mitigate information loss caused by downsampling in the pyramid‐structured encoder and provide semantic compensation to the decoder. This mechanism has substantially improved segmentation performance across various vision applications, including medical image analysis, autonomous driving, and scene understanding.

Despite its success, the encoder–decoder architecture often entails considerable model complexity in practice. Specifically, to achieve improved segmentation performance, some UNet–based models attempt to enhance encoding capacity by designing sophisticated feature fusion mechanisms within the encoder, whereas others focus on innovations in the decoding process, and some address both components. These approaches inevitably increase the number of model parameters and computational cost.

In particular, recent state‐of‐the‐art models have become increasingly complex, such as UNet++,^6^ DenseUNet,^7^ TransUNet,^3^ PolypPVT,^8^ TransCASCADE,^9^ HC‐Mamba,^10^ PC‐UNet,^11^ ES‐UNet,^12^ and PAG‐UNet,^13^ making them unsuitable for deployment in real‐ time or resource‐constrained scenarios. These challenges motivate the exploration of more lightweight architectures that achieve competitive segmentation performance while reducing model complexity and design effort.

**FIGURE 1:**
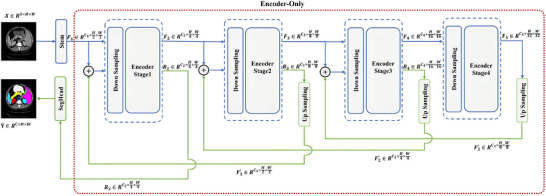
Overview of the proposed decoder‐free medical image segmentation network architecture, R3Net. The core of R3Net is the proposed recursive residual refinement (R3) mechanism, which progressively enhances feature representation capacity through a recursive encoding process while eliminating the need for a separate decoder. Each encoder stage (e.g., Encoder Stage 1) corresponds to an encoding stage of an existing feature extractor (e.g., ResNet).

In this paper, we propose R3Net, a simple yet efficient segmentation framework characterized by a strong encoder and the omission of the traditional bulky decoder. Accordingly, R3Net can be regarded as an encoder‐only, or decoder‐free, architecture for medical image segmentation. Its encoder incorporates a novel recursive residual refinement (R3) mechanism. As illustrated in Figure 1, the R3 mechanism performs feature extraction and refinement in a gradual, recursive manner, ultimately producing high‐resolution dense predictions through a standard segmentation head. Specifically, in the proposed framework, the traditional decoder scheme with skip connections is discarded, and the redesigned encoder focuses on hierarchically capturing multiscale visual representations and progressively fusing them through recursion. Importantly, the encoder can be conveniently implemented by leveraging off‐the‐shelf components for visual representation learning, such as pretrained blocks and modules in ResNet,^14^ Swin Transformer,^15^ or VMamba.^16^ To validate the proposed approach, we conducted extensive experiments on three medical imaging modalities. The experimental results demonstrate that the decoder‐free design not only reduces the number of model parameters and computational cost but also achieves competitive segmentation performance.

## 2 RELATED WORK

The encoder–decoder network architecture is a widely adopted paradigm in various computer vision tasks. In medical imaging, accurate segmentation of anatomical structures and tumors is particularly important for clinical applications such as disease diagnosis, treatment planning, and radiotherapy guidance. Accordingly, encoder–decoder architectures have become a prevailing framework in the image segmentation community. Representative encoders generally fall into four categories: (i) **Convolutional neural network (CNN)‐based backbones**, such as VGG,^17^ ResNet,^14^ DenseNet,^7^ MobileNet,^18^ EfficientNet,^19^ and ConvNeXt,^20^ which naturally achieve pyramid‐like representation learning through spatial downsampling and channel expansion; (ii) **Transformer‐based networks**, such as Vision Transformer (ViT),^21^ Swin Transformer,^15^ Data‐efficient Image Transformers (DeiT),^22^ and Pyramid Vision Transformer (PVT).^23^ Vanilla ViT^21^ and DeiT^22^ lack an inherent multiscale structure because they process flattened image patches through global attention, whereas Swin Transformer^15^ and PVT^23^ explicitly introduce hierarchical architectures to facilitate dense prediction; (iii) **Mamba‐based feature**

**extractors**, such as ViM^24^ and VMamba,^16^ which leverage a selective state‐space modeling mechanism to efficiently capture long‐range dependencies; and (iv) **Hybrid structure encoders**, such as ConvFormer,^25^ CoaT,^26^ and MambaVision,^27^ which combine convolutional inductive biases with attention‐ or state‐space‐based mechanism to balance local feature extraction and global context modeling.

In recent years, increasing efforts have focused on strengthening the encoder components while simplifying their associated decoders. For example, BiSeNet^28^ introduces a spatial and context path to enhance encoding capacity while reducing the complexity of its decoder. HRNet^29^ proposes parallel multi‐resolution branches in its encoder to preserve robust high‐resolution features while reducing decoding overhead. Some Transformer‐based models, such as SegFormer^30^ and SETR,^31^ further simplify their decoders by performing multiscale feature fusion operations exclusively within the encoder. The notable SAM^32^ adopts a heavy encoder and a lightweight decoder, and several recent studies have explored its application in medical image analysis tasks.^33^, ^34^, ^35^, ^36^ Although SAM has demonstrated strong generalization through large‐scale pretraining, the present work focuses on a lightweight architectural mechanism for converting typical UNet–style pipelines into decoder‐free variants. Therefore, the experiments primarily adopt controlled comparisons with the corresponding original baselines to isolate the effect of the proposed R3 mechanism.

Overall, these studies emphasize building stronger encoders to handle feature extraction and fusion, thereby obtaining more effective task‐specific feature representations, while their decoders are correspondingly simplified. Nevertheless, their network architectures have become increasingly complex, rendering them unsuitable for certain real‐time or resource‐constrained scenarios because of their parameter scale and computational cost. This limitation motivates the investigation of whether competitive segmentation performance can be achieved using only an encoder. Accordingly, unlike existing works, this study explores a new segmentation architecture that removes the traditional decoding component and employs a simple, lightweight encoder to achieve the extraction and refinement of multiscale features.

Unlike existing studies that retain explicit decoders while simplifying their design, this work further investigates whether decoding‐related functions can be implicitly realized through recursive encoder reuse within an encoder‐only architecture.

## 3 PROPOSED METHOD

In this section, we present R3Net, a novel medical image segmentation network that operates without a traditional decoder or skip connections. Its core component is the recursive residual refinement (R3) mechanism, which establishes an encoder‐only architecture. In this study, “decoder‐free” refers to the removal of dedicated decoder modules, while resolution recovery and multiscale interaction are implicitly realized through recursive encoder reuse and residual refinement. By eliminating the explicit decoder employed in conventional UNet–style models, R3Net achieves a lightweight segmentation design that controls model complexity while maintaining high segmentation performance. The overall architecture is illustrated in Figure 1.

### 3.1 Hierarchical multiscale feature extraction

To facilitate the development of specific segmentation models based on R3Net, the proposed architecture adopts a hierarchical feature extraction strategy to construct its encoder. Specifically, R3Net is agnostic to the choice of feature extractor and can seamlessly integrate a wide range of off‐the‐shelf CNN‐, Transformer‐, or Mamba‐based backbones to generate hierarchical multiscale feature representations.

As shown in Figure 1, the hierarchical features are formally denoted as a set of visual representations at different scales, i.e., $\left\{F_{1},F_{2},F_{3},F_{4},F_{5}\right\}$, characterized by progressively decreasing spatial resolutions and increasing semantic abstraction. Specifically, given an input image $X\in R^{3\times H\times W}$ fed into a visual feature extractor (e.g., ResNet^14^, Swin Transformer^15^, or VMamba^16^), which comprises multiple encoding stages that perform spatial downsampling and channel expansion, the lowest‐level feature representation $F_{1}\in \;R^{C_{1}\times \frac{H}{2}\times \frac{W}{2}}$ preserves fine‐grained spatial details, whereas the highest‐level feature representation $F_{5}\in R^{C_{5}\times \frac{H}{32}\times \frac{W}{32}}$ encodes high‐level image semantics. These hierarchical multiscale features form the foundation of the proposed recursive residual refinement mechanism.

### 3.2 Recursive residual refinement mechanism

As the core component of R3Net, the proposed R3 mechanism progressively fuses and refines multiscale visual representations by recursively reusing the corresponding encoding stages of an off‐the‐shelf feature extractor with residual fusion pathways. This design enhances feature representation capacity and generates high‐resolution feature maps for final segmentation.

Figure 1 illustrates the recursive process. Specifically, given a higher‐level feature $R_{l}$(e.g., *l* = 4) in the encoder‐only architecture, it is first upsampled and added to the output of a lower‐level encoder stage, i.e., *F_l_*_–__2_.The result of this addition is then processed by the corresponding encoder stage:
(1)

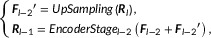




Here, UpSampling(·) consists of two bilinear interpolation operations, each followed by a depthwise separable convolution, achieving a fourfold increase in spatial resolution while aligning the channel dimensions. This design ensures efficient spatial alignment with minimal computational overhead. The function EncoderStage*_l_*_–__2_(·) denotes the (*l* – 2)^th^ stage of the selected backbone. The symbol “+” represents element‐wise addition for residual fusion. To evaluate its impact, additive fusion is compared with concatenation‐based fusion in Section 4.5.3.

Notably, recursive refinement injects high‐level features into the encoder stage two levels below, rather than into the immediately preceding stage. This strategy prevents repeated operations on the deepest stage and enables efficient propagation of high‐level semantics to earlier high‐resolution features. The effect of recursion depth is further analyzed through ablation experiments that vary the number of recursive stages, as presented in Section 4.5.3.

### 3.3 Decoder‐free top‐down refinement pipeline

In the proposed decoder‐free network architecture, R3Net treats its highest‐level feature, i.e., *F*_5_, as the top‐level visual representation, which is obtained from the output of EncoderStage_4_ (·). Specifically, the top‐level representation is defined as *R*_5_, where *R*_5_ = *F*_5_, and it serves as the input to the R3 mechanism. Formally, starting from the top‐level feature *F*_5_, three recursive refinement steps are defined as follows:

(2)

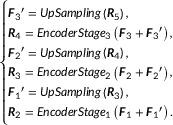




Through the above top‐down refinement process, the output of EncoderStage1 (·), namely *R*_2_, becomes the final encoding result of the encoder‐only architecture, aggregating multi‐level visual semantics and fine‐grained spatial details throughout the recursive encoding pipeline. Subsequently, a lightweight segmentation head is applied to generate the final prediction:

(3) $$\begin{array}{c} \hat{Y}=\mathrm{SegH}\mathrm{ead}\left(R_{2}\right), \end{array}$$

where SegHead(·) denotes the segmentation head network. In practice, SegHead(·) adopts a similar scheme of upsampling and depthwise separable convolution operations as the aforementioned UpSampling(·).

### 3.4 Design rationale

The R3 mechanism comprises three key components: recursive encoding, residual fusion, and feature alignment through upsampling. Recursive encoding propagates high‐level semantic information to earlier stages, thereby refining shallow representations using deeper contextual information. Residual fusion injects refined features through element‐wise addition, preserving the original feature structure and stabilizing gradient flow. Bilinear interpolation followed by depthwise separable convolution aligns spatial resolution and channel dimensions efficiently while maintaining minimal computational overhead. Together, these components form a lightweight recursive refinement pipeline that enables effective multiscale feature interaction without a conventional decoder.

## 4 EXPERIMENTS

### 4.1 Datasets

We evaluated the proposed method on three medical imaging modalities: magnetic resonance imaging (MRI), computed tomography (CT), and ultrasound. The first two modalities are widely used for benchmarking medical image segmentation algorithms. Specifically, the datasets include the Automated Cardiac Diagnosis Challenge (ACDC)^37^ dataset and the Synapse abdominal multi‐organ segmentation dataset (Synapse).^38^ The third modality comprises ultrasound images from the Thyroid Nodule Multimodal Learning (TN‐ML) dataset,^39^ which contains video‐derived frames with expert‐annotated nodule masks and biopsy‐confirmed pathology reports.

#### 4.1.1 ACDC dataset

The ACDC^37^ dataset was released for the ACDC and consists of cardiac MRI scans from multiple patients. Each patient scan is manually annotated with ground truth labels for three anatomical structures: the left ventricle, right ventricle, and myocardium. Following TransUNet,^3^ 70 cases were used for training, 10 for validation, and 20 for testing.

#### 4.1.2 Synapse dataset

The Synapse^38^ dataset contains clinical CT images from 30 patients for abdominal multi‐organ segmentation, totaling 3,779 axial contrast‐enhanced images. The images have a uniform resolution of 512 × 512 pixels and provide accurate annotations of organ contours. Following TransUNet,^3^ the dataset was randomly divided into 18 cases for training and 12 cases for testing. The experiments focused on the segmentation of 8 abdominal organs: the aorta, gallbladder, left kidney, right kidney, liver, pancreas, spleen, and stomach.

#### 4.1.3 TN‐ML dataset

The TN‐ML dataset^39^ was derived from a previous study on computer‐aided diagnosis of thyroid nodules and comprises 580 thyroid ultrasound videos collected from different patients, along with their corresponding pathological reports. Each video was temporally sampled to extract eight representative frames, and each frame was paired with a nodule‐level segmentation mask annotated by experienced clinicians according to biopsy‐confirmed pathology, yielding a total of 4,640 labeled ultrasound frames.

Among the annotated nodules, 374 cases were malignant and 206 cases were benign according to biopsy‐confirmed pathology reports, enabling subtype‐level evaluation of segmentation performance across different pathological categories.

**FIGURE 2:**
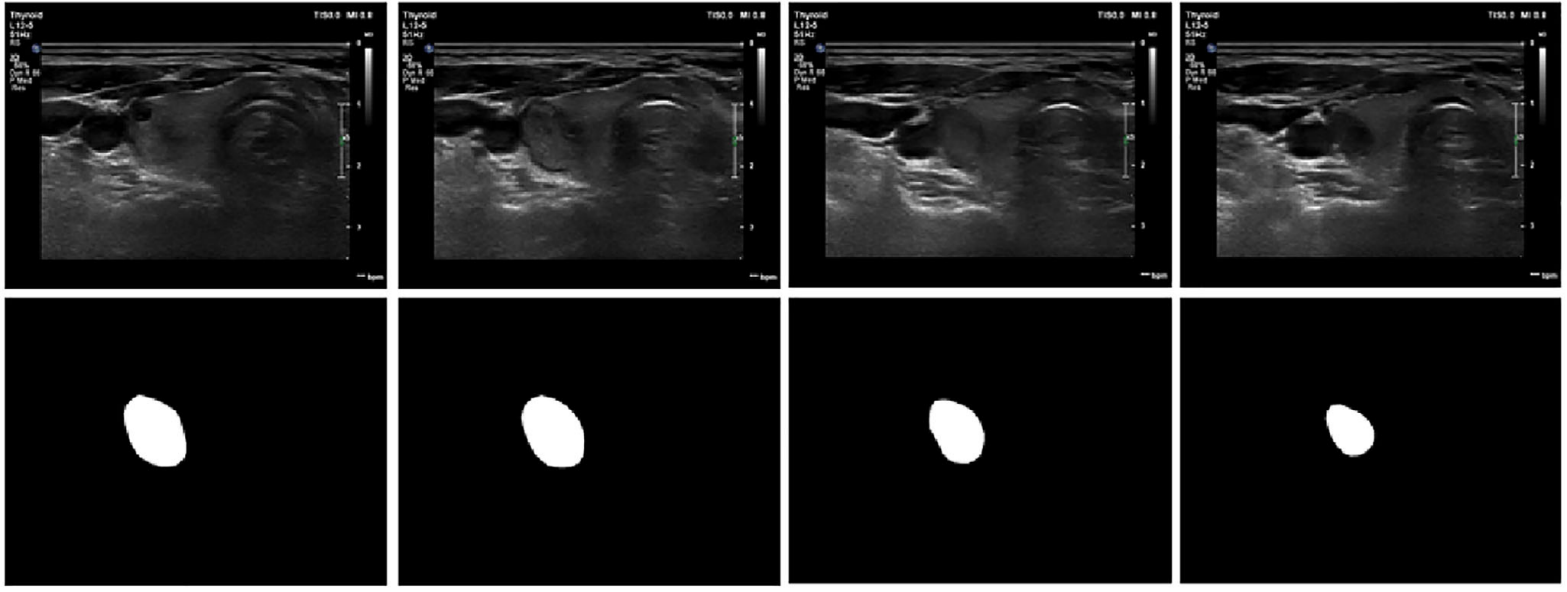
A sequence of nodular segmentation masks corresponding to four consecutive frames sampled from a thyroid ultrasound video.

To avoid potential data leakage caused by the high correlation among frames from the same video sequence, the dataset was split in a patient‐wise manner into training, validation, and test sets at a ratio of 70%, 10%, and 20%, respectively. This split corresponded to 406 videos (3,248 frames) for training, 58 videos (464 frames) for validation, and 116 videos (928 frames) for testing. Videos from the same patient were assigned exclusively to a single subset. Representative examples are shown in Figure 2.

Importantly, the dataset includes pathology‐confirmed malignant nodules. Accurate delineation of these lesions is clinically relevant for tumor localization and oncological imaging analysis. Reliable segmentation of malignant nodules may facilitate downstream tasks such as lesion assessment, treatment planning, and image‐guided clinical decision‐making in tumor‐related imaging workflows.

### 4.2 Evaluation metrics

To quantitatively evaluate segmentation performance, five commonly used evaluation metrics were adopted: the Dice similarity coefficient (DSC), mean intersection over union (mIoU), the 95th percentile Hausdorff distance (HD95), sensitivity, and specificity.

For multi‐class segmentation tasks, sensitivity and specificity are computed in a one‐vs‐rest manner for each class and then macro‐averaged across all classes.

For all datasets, segmentation performance is evaluated on a slice‐ or frame‐level basis, and the final results are obtained by averaging the metrics across all test slices or frames.

The DSC is defined as:

(4) $$\begin{array}{c} \mathrm{DSC}=\frac{2\mathrm{TP}}{2\mathrm{TP}+\mathrm{FP}+\mathrm{FN}}, \end{array}$$

where TP, TN, FP, and FN denote the numbers of true‐positive, true‐negative, false‐positive, and false‐negative pixels, respectively.

The intersection over union (IoU) is defined as:

(5) $$\begin{array}{c} \mathrm{IoU}=\frac{\mathrm{TP}}{\mathrm{TP}+\mathrm{FP}+\mathrm{FN}}. \end{array}$$

The mIoU is obtained by averaging IoU across all target classes:

(6) $$\begin{array}{c} \mathrm{mIoU}=\frac{1}{K}\sum_{k=1}^{K}\mathrm{Io}U_{k}, \end{array}$$

where *K* denotes the number of classes.

Sensitivity measures the proportion of correctly identified positive pixels:

(7) $$\begin{array}{c} \mathrm{Sens}\mathrm{itiv}\mathrm{ity}=\frac{\mathrm{TP}}{\mathrm{TP}+\mathrm{FN}}. \end{array}$$

Specificity measures the proportion of correctly identified negative pixels:

(8) $$\begin{array}{c} \mathrm{Spec}\mathrm{ific}\mathrm{ity}=\frac{\mathrm{TN}}{\mathrm{TN}+\mathrm{FP}}. \end{array}$$

To evaluate boundary accuracy, HD95 is employed. It measures the distance between the predicted boundary and ground‐truth boundary while reducing the influence of outliers:

(9) $$\begin{array}{c} \mathrm{HD}95\left(A,B\right)=\max\left\{\mathrm{perc}\mathrm{entil}e_{95}\left(d\left(A,B\right)\right),\mathrm{perc}\mathrm{entil}e_{95}\left(d\left(B,A\right)\right)\right\}, \end{array}$$

where *A* and *B* denote the sets of boundary points from the predicted segmentation and the ground truth, respectively, and d(·) represents the Euclidean distance.

Higher values of DSC, mIoU, sensitivity, and specificity indicate better segmentation performance, whereas lower HD95 values indicate more accurate boundary delineation.

### 4.3 Implementation details

The proposed R3Net was implemented using the PyTorch framework (version 2.1.1), and all experiments were conducted on a single NVIDIA GeForce RTX 3090 GPU. During training, standard data augmentation strategies were applied, including resizing input images to 224 × 224 pixels, horizontal and vertical flipping, random rotation, Gaussian noise injection, Gaussian blurring, and contrast adjustment.

The network was optimized using the AdamW optimizer with a batch size of 32 for 300 epochs. The initial learning rate was set to 5 × 10^−4^ and updated using a cosine annealing schedule. To mitigate overfitting, different weight decay coefficients were adopted for different datasets: 1 × 10^−4^ for the ACDC and TN‐ML datasets and 1 × 10^−3^ for the Synapse dataset.

For model optimization, a hybrid loss function combining Dice loss and cross‐entropy (CE) loss was employed:

(10) $$\begin{array}{c} \begin{array}{c} L_{\mathrm{total}}=\alpha L_{\mathrm{Dice}}+\left(1-\alpha \right)L_{\mathrm{CE}} \end{array}, \end{array}$$

where α controls the balance between region overlap and pixel‐wise classification accuracy. The loss was applied to the final prediction Y^. In all experiments, α was set to 0.5.

For reproducibility, preprocessing procedures and computational cost settings are specified as follows. All images were resized to 224 × 224 pixels using cubic interpolation implemented with a third‐order spline. In addition, the number of parameters and floating‐point operations (FLOPs) were computed using the calflops tool with an input tensor of shape (1, 3, 224, 224), corresponding to a batch size of one. The computation was performed under default floating‐point precision without mixed‐precision acceleration. Convolutional layers were counted following the standard multiply–accumulate convention, consistent with the default implementation of the tool.

For a fair and controlled comparison, all baseline models, including UNet, Swin‐UNet, and VM‐UNet, were retrained using the same data split, optimization settings, and training protocol as R3Net, rather than their originally reported configurations. Therefore, the reported differences primarily reflect architectural design rather than variations in training strategy.

Based on the above settings, the proposed R3 mechanism was first evaluated on two public medical image segmentation benchmarks and subsequently assessed for its practical value on the clinical TN‐ML ultrasound dataset.

### 4.4 Comparative study on public benchmarks

To evaluate the effectiveness and generalizability of the proposed R3 mechanism, comparative experiments were first conducted on two public medical image segmentation benchmarks, ACDC and Synapse. Three representative encoder–decoder segmentation models were selected for comparison.

Three representative encoder–decoder segmentation models, including UNet, Swin‐UNet, and VM‐UNet, were selected as baselines. Correspondingly, three R3‐based variants were constructed using ResNet, Swin Transformer, and VMamba as backbone feature extractors, denoted as R3Net‐CNN, R3Net‐Swin, and R3Net‐VM, respectively. These controlled paired comparisons were designed to isolate the effect of the proposed recursive residual refinement mechanism under consistent training settings.

#### 4.4.1 Performance on the ACDC dataset

Table 1 summarizes the quantitative results on the ACDC dataset. Overall, the proposed R3‐based models achieved comparable or improved segmentation performance while consistently reducing model complexity compared with their corresponding encoder–decoder baselines.

**TABLE 1:** Performance comparison on the ACDC dataset. The best results within each backbone pair are highlighted in bold.

Methods	Decoder	R3	#Params (M) ↓	#FLOPs (G) ↓	DSC (%) ↑	mIoU (%) ↑	HD95 (mm) ↓	Sensitivity (%) ↑	Specificity (%) ↑
UNet	✓		31.04	73.86	91.02	84.10	1.92	91.96	99.89
R3Net‐CNN		✓	**19.74**	**48.22**	**91.35**	**84.52**	**1.71**	**92.07**	**99.90**
Swin‐UNet	✓		27.17	11.81	91.47	84.73	1.80	92.12	**99.89**
R3Net‐Swin		✓	**22.56**	**9.67**	**91.56**	**84.85**	**1.69**	**92.90**	**99.89**
VM‐UNet	✓		27.43	8.16	91.64	85.02	**1.41**	92.33	**99.90**
R3Net‐VM		✓	**15.79**	**7.12**	**91.92**	**85.43**	1.79	**92.78**	**99.90**

For the CNN‐based architecture, R3Net‐CNN achieved a Dice score of 91.35%, compared with 91.02% obtained by UNet. At the same time, the number of parameters was reduced from 31.04 M to 19.74 M, and the computational cost decreased from 73.86 G to 48.22 G FLOPs. Improvements were also observed in other evaluation metrics, including an increase in mIoU (84.52% vs. 84.10%) and a reduction in HD95 (1.71 mm vs. 1.92 mm). Sensitivity and specificity showed small but consistent improvements, indicating that the proposed recursive refinement mechanism enhanced segmentation accuracy while maintaining computational efficiency.

For the Transformer‐based architecture, R3Net‐Swin slightly improved the Dice score from 91.47% to 91.56% and increased mIoU from 84.73% to 84.85%. Meanwhile, the parameter count decreased by 4.61 M and FLOPs decreased by 2.14 G. A modest reduction in HD95 (1.69 mm vs. 1.80 mm) further suggested improved boundary delineation.

For the Mamba‐based architecture, R3Net‐VM achieved the highest Dice score of 91.92% and an mIoU of 85.43%, while using only 15.79 M parameters and 7.12 G FLOPs. Although HD95 was slightly higher than that of VM‐UNet, the proposed model maintained competitive boundary accuracy with substantially reduced model complexity.

**FIGURE 3:**
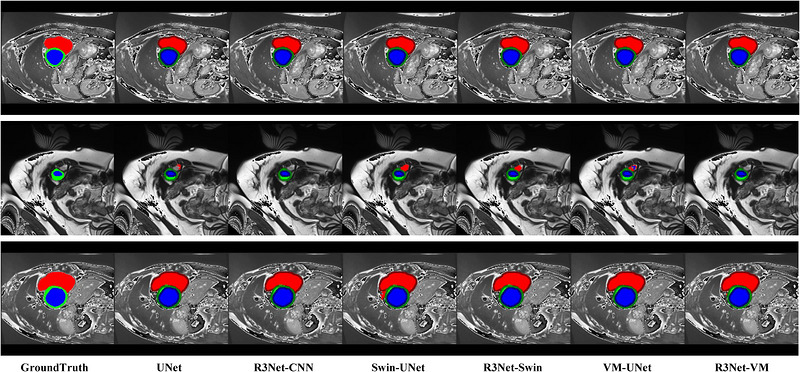
Visual comparison of different methods on the ACDC dataset. The first column shows the ground truth, and the subsequent columns display the segmentation predictions of three classical baselines and their corresponding R3‐enhanced variants, respectively. All three R3‐enhanced models achieve segmentation results that are comparable to their respective baseline models.

Qualitative comparisons are shown in Figure 3. The R3‐based models generated segmentation results that were visually comparable to those of their corresponding baselines, suggesting that accurate dense prediction could be maintained without an explicit decoder structure.

To further examine whether this observation generalized to a more challenging multi‐organ segmentation setting, the proposed models were next evaluated on the Synapse dataset.

#### 4.4.2 Performance on the Synapse dataset

Table 2 summarizes the quantitative results on the Synapse dataset. Overall, the proposed R3‐based models substantially reduced model size and computational cost while maintaining competitive segmentation performance across different backbone architectures.

**TABLE 2:** Performance comparison on the Synapse dataset. The best results within each backbone pair are highlighted in bold.

Methods	Decoder	R3	#Params (M) ↓	#FLOPs (G) ↓	DSC (%) ↑	mIoU (%) ↑	HD95 (mm) ↓	Sensitivity (%) ↑	Specificity (%) ↑
UNet	✓		31.04	73.89	82.09	72.76	34.20	80.97	98.91
R3Net‐CNN		✓	**19.74**	**48.28**	**82.19**	**73.03**	**24.70**	**82.18**	**98.92**
Swin‐UNet	✓		27.17	11.86	**78.55**	**68.55**	**26.27**	**78.06**	**98.90**
R3Net‐Swin		✓	**22.56**	**9.72**	77.76	67.09	35.49	77.51	**98.90**
VM‐UNet	✓		27.43	8.17	**83.18**	**73.89**	**20.38**	**82.72**	**98.91**
R3Net‐VM		✓	**15.79**	**7.14**	81.85	72.05	25.08	81.26	**98.91**

For the CNN‐based architecture, R3Net‐CNN achieved a Dice score of 82.19%, slightly higher than that of UNet (82.09%). Meanwhile, the number of parameters was reduced from 31.04 M to 19.74 M, and the computational cost decreased from 73.89 G to 48.28 G FLOPs. Improvements were also observed in other metrics, including an increase in mIoU (73.03% vs. 72.76%), a notable reduction in HD95 (24.70 mm vs. 34.20 mm), and higher sensitivity, indicating improved boundary delineation and detection reliability.

For the Transformer‐based architecture, R3Net‐Swin reduced the parameter count from 27.17 M to 22.56 M and decreased FLOPs from 11.86 G to 9.72 G. Although the Dice score decreased slightly (77.76% vs. 78.55%), the model maintained comparable segmentation performance while achieving substantially lower computational cost.

For the Mamba‐based architecture, R3Net‐VM significantly reduced model size (15.79 M vs. 27.43 M parameters) and computational cost (7.14 G vs. 8.17 G FLOPs). Although the Dice score decreased compared with VM‐UNet (81.85% vs. 83.18%), the overall performance remained competitive considering the substantial reduction in model complexity.

**FIGURE 4:**
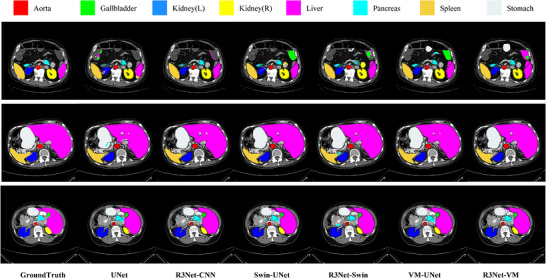
Visual comparison of different methods on the Synapse dataset. The first column shows the ground truth, and the subsequent columns display the segmentation predictions of three classical baselines and their corresponding R3‐enhanced variants, respectively. The three R3‐enhanced models achieve segmentation performance comparable to their respective baseline models.

Qualitative comparisons are presented in Figure 4. The visual results were generally consistent with the quantitative findings in Table 2. Although minor differences were observed in certain regions, the R3‐based models produced segmentation maps that were broadly comparable to those of the baseline models while substantially reducing model complexity.

These results further suggest that the effectiveness of the proposed R3 mechanism may depend on the spatial characteristics of the underlying feature extractor, which are analyzed in greater detail below.

#### 4.4.3 Impact of feature extractors on the effectiveness of the R3 mechanism

ResNet, Swin Transformer, and VMamba are widely used backbone networks for visual feature extraction. Compared with ResNet, both Swin Transformer and VMamba adopt non‐overlapping patch partitioning in the early stages of the network, which reduces the spatial resolution of the input image from 224 × 224 to 56 × 56 at an early stage. Such aggressive early downsampling may limit the effectiveness of the proposed residual refinement mechanism. Specifically, the key residual fusion in R3Net is designed to operate at a spatial resolution of 112 × 112. However, this intermediate resolution is not preserved in Swin Transformer and VMamba because of their patch‐based downsampling strategy.

As shown in Table 2, this architectural characteristic was associated with a slight decrease in segmentation accuracy for R3Net‐Swin and R3Net‐VM, with Dice score reductions of 0.79% and 1.33%, respectively. Nevertheless, both models achieved substantial reductions in parameter size and computational cost.

These observations suggest that the R3 mechanism is more effective when higher‐resolution intermediate features are preserved during feature extraction. This property is more commonly observed in CNN‐based architectures than in patch‐based models.

Motivated by these observations, the proposed approach was further evaluated on a clinical thyroid ultrasound dataset to assess its practical value under more challenging real‐world imaging conditions.

### 4.5 Case study on the TN‐ML dataset

To further assess the practical value of the proposed R3 mechanism under real‐world clinical imaging conditions, a case study was conducted on the TN‐ML dataset, a thyroid ultrasound dataset derived from a prior intelligent diagnosis system.^39^ Compared with public MRI and CT benchmarks, this dataset presents additional challenges, such as low contrast, speckle noise, and irregular lesion boundaries, thereby providing a more clinically relevant test bed for evaluating the proposed decoder‐free architecture. In particular, the dataset contains biopsy‐confirmed malignant nodules, making it a tumor‐related imaging scenario for evaluating segmentation algorithms in clinically meaningful settings.

#### 4.5.1 Segmentation challenges on the TN‐ML dataset

Unlike the two public medical imaging modalities described above (CT and MRI), all imaging data in the TN‐ML dataset were acquired from routine thyroid ultrasound examinations performed by clinicians. These ultrasound video data are characterized by low image quality, high speckle noise, low contrast, high tissue similarity, motion artifacts, and temporal continuity. Such properties pose substantial challenges to accurate thyroid nodule segmentation, making this task particularly difficult in real‐world clinical scenarios. These characteristics render the TN‐ML dataset a challenging yet clinically meaningful benchmark for evaluating whether the proposed R3 mechanism can maintain segmentation quality under realistic ultrasound conditions. Representative examples of these challenges are shown in the first row of Figure 2.

#### 4.5.2 Evaluation on Thyroid Ultrasound Videos

Table 3 presents the quantitative results on the TN‐ML dataset. Overall, the proposed R3‐based models achieved comparable or slightly improved segmentation performance while substantially reducing model complexity compared with their corresponding encoder–decoder baselines.

**TABLE 3:** Performance comparison on the TN‐ML dataset. The best results within each backbone pair are highlighted in bold.

Methods	Decoder	R3	#Params (M) ↓	#FLOPs (G) ↓	DSC (%) ↑	mIoU (%) ↑	HD95 (mm) ↓	Sensitivity (%) ↑	Specificity (%) ↑
UNet	✓		31.04	73.89	77.59	63.22	16.83	81.25	88.63
R3Net‐CNN		✓	**19.74**	**48.28**	**77.83**	**63.65**	**15.27**	**81.89**	**88.71**
Swin‐UNet	✓		27.17	11.86	80.34	67.08	**13.80**	84.12	89.35
R3Net‐Swin		✓	**22.56**	**9.72**	**80.40**	**67.23**	14.31	**84.37**	**89.42**
VM‐UNet	✓		27.43	8.17	80.46	67.32	14.06	84.56	89.51
R3Net‐VM		✓	**15.79**	**7.14**	**80.53**	**67.45**	**13.97**	**84.79**	**89.58**

For the CNN‐based architecture, R3Net‐CNN achieved a Dice score of 77.83% and an mIoU of 63.65%, surpassing the standard UNet. Model complexity was significantly reduced, with the number of parameters decreasing from 31.04 M to 19.74 M and the computational cost measured in FLOPs decreasing from 73.89 G to 48.28 G. Improvements were also observed in boundary delineation and detection reliability, as reflected by HD95, sensitivity, and specificity.

In the Transformer‐based architecture, R3Net‐Swin slightly improved the Dice score to 80.40% and the mIoU to 67.23% relative to Swin‐UNet. Simultaneously, the number of parameters was reduced to 22.56 M and FLOPs decreased to 9.72 G. Although HD95 increased modestly, the model retained competitive segmentation performance while benefiting from substantially lower computational cost.

For the Mamba‐based architecture, R3Net‐VM achieved a Dice score of 80.53% and an mIoU of 67.45% with only 15.79 M parameters and 7.14 G FLOPs. Boundary accuracy and detection metrics, including HD95, sensitivity, and specificity, remained comparable to VM‐UNet, indicating that the recursive refinement mechanism improved computational efficiency without compromising segmentation quality.

A more detailed analysis was conducted by examining benign and malignant nodules separately, as summarized in Table 4. The pathology labels were defined at the nodule level, whereas the segmentation metrics were computed on a frame basis and then aggregated within each pathological category. For benign nodules, R3Net‐CNN achieved a Dice score of 78.17% and an mIoU of 64.22%, with an HD95 of 15.38 mm. For malignant nodules, which often exhibit more irregular shapes and blurred boundaries in ultrasound imaging, the model achieved a Dice score of 77.39% and an mIoU of 63.01%, with an HD95 of 16.75 mm.

**TABLE 4:** Segmentation performance on the TN‐ML dataset for benign and malignant thyroid nodules. The best results within each nodule‐type pair are highlighted in bold.

Method	Nodule Type	DSC (%) ↑	mIoU (%) ↑	HD95 (mm) ↓	Sensitivity (%) ↑	Specificity (%) ↑
UNet	Benign	77.81	63.77	16.91	81.50	88.75
R3Net‐CNN	Benign	**78.17**	**64.22**	**15.38**	**82.03**	**88.96**
UNet	Malignant	77.24	62.86	**15.25**	81.17	88.53
R3Net‐CNN	Malignant	**77.39**	**63.01**	16.75	**81.54**	**88.67**

Although malignant nodules generally presented more challenging boundary characteristics, the performance gap between the two pathological categories remained relatively small. This observation suggests that the proposed recursive residual refinement mechanism maintained stable segmentation capability across different nodule types. In addition, sensitivity and specificity remained consistently high for both benign and malignant lesions, indicating reliable detection and delineation performance in clinically heterogeneous scenarios.

**FIGURE 5:**
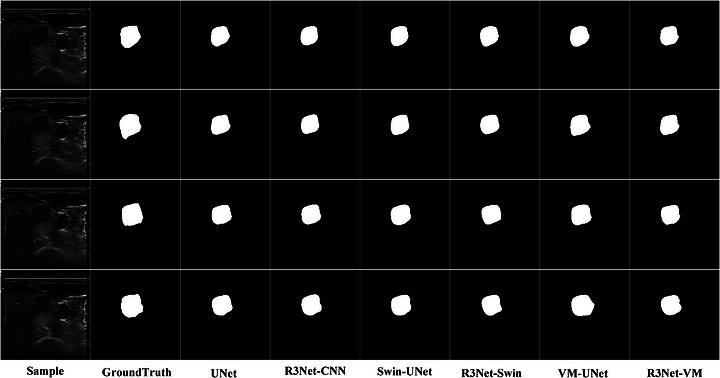
Visualization of qualitative comparison results with classical state‐of‐the‐art segmentation models on a thyroid ultrasound video sampled at four frames. The example illustrates several challenges in thyroid nodule segmentation, including low image quality, speckle noise, low contrast, tissue similarity, and irregular boundaries.

As shown in Figure 5, the visual comparisons further supported these findings. The segmentation maps generated by the R3‐based models were comparable to those produced by the baseline models, confirming that accurate dense prediction could be achieved without an explicit decoder.

To further understand which design choices contributed to these performance gains, ablation experiments were subsequently conducted on the CNN‐based variant of R3Net.

#### 4.5.3 Ablation study on R3Net‐CNN

To better understand the contribution of the proposed R3 mechanism, ablation experiments were conducted on the TN‐ML dataset using the CNN‐based backbone. Specifically, two aspects were investigated: (i) the feature fusion strategy employed in residual refinement and (ii) the recursive refinement depth of the R3 mechanism. These experiments clarified how hierarchical feature interaction and iterative refinement influenced overall segmentation performance.

##### 4.5.3.1 Fusion strategy

A crucial component in R3Net‐CNN is the method used to fuse multiscale features. Two commonly adopted fusion schemes were compared: *element‐wise addition (Add)* and *channel‐wise concatenation (Concat)*. As shown in Table 5, Add consistently outperformed Concat across all evaluation metrics while also reducing model complexity. Specifically, Add improved DSC from 76.29% to 77.83% and mIoU from 61.35% to 63.65%, while reducing HD95 from 16.30 mm to 15.27 mm. Meanwhile, the parameter count and FLOPs decreased from 19.91 M to 19.74 M and from 51.60 G to 48.28 G, respectively. Sensitivity and specificity were also slightly improved. These results indicate that element‐wise addition provides a more compact and effective feature integration scheme for recursive refinement in R3Net‐CNN.

**TABLE 5:** Comparison of different fusion strategies in R3Net‐CNN on the TN‐ML dataset. The best results are highlighted in bold.

Fusion Type	#Params (M) ↓	FLOPs (G) ↓	DSC (%) ↑	mIoU (%) ↑	HD95 (mm) ↓	Sensitivity (%) ↑	Specificity (%) ↑
Concat	19.91	51.60	76.29	61.35	16.30	80.52	88.57
Add	**19.74**	**48.28**	**77.83**	**63.65**	**15.27**	**81.89**	**88.71**

##### 4.5.3.2 Recursive Depth

The effect of recursive refinement depth was further investigated by varying the number of recursive stages from 0 to 4. Here, *D* denotes the number of times the R3 refinement process is recursively applied, and *D* = 0 indicates that no recursive refinement is performed.

Table 6 reports the segmentation results under different recursive depths. As *D* increased from 0 to 3, DSC improved steadily from 72.46% to 77.83%, and mIoU increased from 56.43% to 63.65%, whereas HD95 decreased from 18.72 mm to 15.27 mm. This progressive improvement indicates that recursive refinement effectively enhanced multiscale feature interaction and boundary delineation. However, when the depth was further increased to *D* = 4, performance declined markedly, with DSC decreasing to 72.39% and HD95 increasing to 18.96 mm. This finding suggests that excessive recursion may introduce redundant feature transformations and optimization difficulty, thereby offsetting the benefits of iterative refinement. Meanwhile, the number of parameters increased only slightly and FLOPs rose moderately as *D* increased, highlighting the favorable efficiency of the proposed design.

**TABLE 6:** Effect of recursive depth on segmentation performance of R3Net‐CNN on the TN‐ML dataset. The stage (*D*) corresponds to the number of recursions of the R3 module. The best results are highlighted in bold.

Stage (*D*)	#Params (M) ↓	FLOPs (G) ↓	DSC (%) ↑	mIoU (%) ↑	HD95 (mm) ↓	Sensitivity (%) ↑	Specificity (%) ↑
0	**19.56**	**29.70**	72.46	56.43	18.72	77.35	88.21
1	**19.56**	35.55	73.78	58.25	17.91	78.62	88.34
2	19.60	41.77	75.81	61.02	16.84	80.13	88.47
3	19.74	48.28	**77.83**	**63.65**	**15.27**	**81.89**	**88.71**
4	20.27	54.67	72.39	58.61	18.96	77.21	88.18

From an empirical perspective, for typical medical image segmentation tasks—especially CT, MRI, and ultrasound segmentation—we recommend setting the recursive depth of the R3 mechanism to *D* = 3, because excessively deep refinement may introduce feature redundancy, whereas overly shallow refinement may not fully integrate multiscale cues. In addition, element‐wise addition (Add) is generally preferred as the feature fusion method because it provides a favorable balance between computational cost and segmentation accuracy. If improved performance on small targets is required, a lightweight hybrid fusion strategy, such as combining Add with a channel‐reduced Concat, may be worth exploring.

In summary, this analysis confirms that R3Net‐CNN effectively leveraged recursive residual refinement to progressively enhance segmentation quality. Element‐wise addition served as a compact and efficient fusion strategy, whereas the recursive depth should be carefully selected to balance segmentation accuracy and computational cost. These findings provide practical guidance for applying the R3 mechanism to other CNN‐based segmentation architectures.

Beyond segmentation accuracy, practical deployment also depends on hardware‐aware efficiency. Therefore, inference time and GPU memory usage of the proposed R3‐based models were further evaluated on the TN‐ML dataset.

#### 4.5.4 Potential for clinical deployment

As summarized in Table 7, the proposed R3 mechanism consistently improved inference efficiency across all backbones. Specifically, R3Net‐CNN reduced inference time and GPU memory usage relative to UNet, whereas the efficiency gains were more pronounced for the Swin‐ and VMamba‐based variants. These results indicate that the proposed decoder‐free design was not only parameter‐efficient but also hardware‐friendly in practical inference settings.

**TABLE 7:** Inference efficiency comparison of baseline and R3‐based models on the TN‐ML dataset. The best results within each backbone pair are highlighted in bold.

Methods	Decoder	R3	Inference Time (ms) ↓	GPU Memory Usage (GB) ↓
UNetR3Net‐CNN	✓	✓	4.18**4.08**	0.30**0.29**
Swin‐UNetR3Net‐Swin	✓	✓	7.37**5.82**	0.16**0.14**
VM‐UNetR3Net‐VM	✓	✓	7.89**5.98**	0.17**0.12**

From a clinical perspective, such efficiency improvements may facilitate real‐time or near‐real‐time segmentation on standard ultrasound workstations, thereby supporting more efficient visualization of thyroid boundaries during scanning. More broadly, reduced latency and memory usage may lower deployment barriers and improve the feasibility of integrating R3‐based models into existing clinical imaging pipelines.

### 4.6 Training stability of R3Net variants

In addition to segmentation accuracy and efficiency, the optimization stability of the proposed R3‐based models during training was further examined.

**FIGURE 6:**
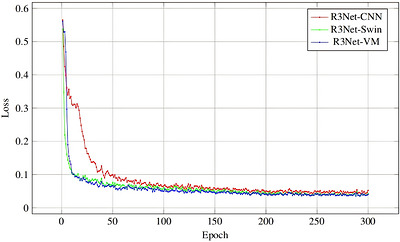
Training loss progression of R3Net‐CNN, R3Net‐Swin, and R3Net‐VM on the ACDC dataset, showing stable convergence. Here, the three R3‐enhanced segmentation models denote those using ResNet, Swin Transformer, and VMamba, respectively, as their backbone feature extractors.

To assess the convergence behavior of the proposed R3Net variants, the training loss over epochs was tracked on the ACDC dataset. As shown in Figure 6, all variants exhibited a steady decrease in training loss, indicating stable optimization and good convergence behavior throughout training. This observation further supports the feasibility of the proposed recursive residual refinement mechanism across different backbone architectures.

## 5 DISCUSSION

This work explored an encoder‐centric segmentation paradigm by replacing conventional decoder modules with a recursive residual refinement (R3) mechanism. By reusing encoder stages for progressive feature enhancement, R3Net reduced architectural redundancy and achieved substantial savings in parameters and computational cost while maintaining competitive segmentation accuracy across multiple medical imaging modalities.

From a practical perspective, the reduced model complexity of R3Net may benefit real‐world clinical workflows in which latency, hardware availability, and system integration are important considerations. For example, in radiotherapy planning, faster and more memory‐efficient contouring models may facilitate interactive refinement by clinicians and shorten the segmentation‐and‐review loop. In ultrasound‐assisted diagnosis, lightweight models are more suitable for real‐time or near‐real‐time inference on standard workstations, enabling on‐the‐fly visualization of lesion boundaries during scanning. More broadly, reducing computational requirements may lower deployment barriers in resource‐constrained environments and simplify integration into existing clinical imaging pipelines.

This study represented an initial exploration of decoder‐free segmentation architectures based on recursive encoder refinement. Several limitations merit discussion. First, the accuracy improvements over strong encoder–decoder baselines were modest in certain settings, and slight performance degradation occurred when R3Net was combined with backbones that employed aggressive early downsampling, in which high‐resolution spatial information was insufficiently preserved for subsequent refinement. This finding suggests that the effectiveness of recursive refinement is related to the spatial characteristics of the underlying encoder. Second, although efficiency improvements were demonstrated in terms of parameters, FLOPs, inference time, and GPU memory usage, more comprehensive hardware‐aware evaluations under broader deployment scenarios would further strengthen the practical assessment. Third, although R3Net was evaluated on established benchmarks and a clinical ultrasound dataset containing malignant thyroid nodules, additional validation on larger multi‐center cohorts and external datasets would help further assess generalizability and robustness.

For clarity, the term “decoder‐free” in R3Net refers to the removal of dedicated decoder modules rather than the elimination of decoding functionality. In this architecture, feature upsampling and multiscale fusion are implicitly realized through recursive encoder reuse and residual refinement, providing a more parameter‐efficient alternative to traditional UNet‐style decoders. Table 8 summarizes the functional differences between explicit decoder structures and the implicit decoding behavior in R3Net.

**TABLE 8:** A concise comparison between the explicit decoder in UNet‐style architectures and the implicit decoding behavior in R3Net.

Function	UNet‐style explicit decoder	R3Net implicit decoding (decoder‐free)
Upsampling / resolution recovery	Progressive upsampling layers in the decoder recover spatial resolution stage by stage.	Upsampling is performed within the recursive refinement pathway and the lightweight segmentation head, without a dedicated decoder module.
Feature fusion across scales	Skip connections fuse encoder features with decoder features (e.g., concatenation or addition) at each scale.	Residual fusion is achieved by element‐wise addition between upsampled refined features and shallower encoder features during recursive refinement.
Semantic recovery / detail refinement	The decoder aggregates high‐level semantics and refines boundaries via multiscale fusion.	High‐level semantics are injected into shallow representations through recursive encoder reuse, producing refined high‐resolution features for segmentation.

Despite these limitations, the primary contribution of R3Net lies in opening a new design space for efficient segmentation architectures. The results suggest that competitive medical image segmentation can be achieved without explicit decoder structures, thereby challenging the conventional assumption of symmetric encoder–decoder designs. Although the experiments focused on controlled paired comparisons against representative baselines to isolate the effect of the proposed R3 mechanism, future work will explore adaptive refinement strategies, partial encoder reuse, and spatial prior integration, and will further evaluate R3Net on broader cohorts and against more recent segmentation approaches to strengthen its empirical positioning.

## 6 CONCLUSION

In this work, R3Net was proposed as a novel decoder‐free architecture for medical image segmentation, which recursively refines multiscale features within the encoder through residual pathways. Extensive experiments demonstrated that R3Net effectively reduced parameter redundancy and architectural complexity while maintaining strong segmentation performance. The proposed lightweight architecture may also be beneficial for tumor delineation tasks in oncological imaging and radiotherapy‐related workflows, in which efficient and reliable segmentation tools are important for supporting clinical image analysis.

## FUNDING STATEMENT

This work was supported in part by the Research and Development Center of Transport Industry of New Generation of Artificial Intelligence Technology (Grant No. 202302H), the Hospital Research Project (Grant No. E010221008), the Sanming Project of Medicine in Shenzhen (Grant No. SZSM201612063), and the Shenzhen Key Medical Discipline Construction Fund (Grant No. SZXK013).

## ETHICAL STATEMENT

The studies involving human participants were reviewed and approved by the institutional review board of the National Cancer Center/National Clinical Research Center for Cancer/Cancer Hospital & Shenzhen Hospital. All methods were carried out in accordance with relevant guidelines and regulations. Informed consent was not required for this retrospective study. Written informed consent from the involved patients was therefore not required.

## CONFLICT OF INTEREST STATEMENT

The authors declare that they have no conflict of interest.

## Data Availability

The data used in this study include both publicly available and non‐public datasets. The Synapse and ACDC datasets are publicly available. The private dataset used in this study is not publicly available because of patient privacy and ethical restrictions but may be made available from the corresponding author upon reasonable request.
